# Synergistic Effect of *Sophora japonica* and *Glycyrrhiza glabra* Flavonoid-Rich Fractions on Wound Healing: *In Vivo* and Molecular Docking Studies

**DOI:** 10.3390/molecules28072994

**Published:** 2023-03-27

**Authors:** Shaza H. Aly, Ahmed M. Elissawy, Abdulla M. A. Mahmoud, Fatma Sa’eed El-Tokhy, Sherif S. Abdel Mageed, Hadia Almahli, Sara T. Al-Rashood, Faizah A. Binjubair, Mahmoud A. El Hassab, Wagdy M. Eldehna, Abd El-Nasser B. Singab

**Affiliations:** 1 Department of Pharmacognosy, Faculty of Pharmacy, Badr University in Cairo (BUC), Badr City, Cairo 11829, Egypt; 2 Department of Pharmacognosy, Faculty of Pharmacy, Ain-Shams University, Cairo 11566, Egypt; 3 Centre of Drug Discovery Research and Development, Ain Shams University, Cairo 11566, Egypt; 4 Pharmacology and Toxicology Department, Faculty of Pharmacy, Badr University in Cairo (BUC), Cairo 11829, Egypt; 5 Department of Pharmaceutics and Pharmaceutical Technology, Badr University in Cairo (BUC), Badr city, Cairo 11829, Egypt; 6 Department of Chemistry, University of Cambridge, Cambridge CB2 1EW, UK; 7 Department of Pharmaceutical Chemistry, College of Pharmacy, King Saud University, P.O. Box 2457, Riyadh 11451, Saudi Arabia; 8 Department of Medicinal Chemistry, Faculty of Pharmacy, King Salman International University (KSIU), South Sinai 46612, Egypt; 9 Department of Pharmaceutical Chemistry, Faculty of Pharmacy, Kafrelsheikh University, Kafrelsheikh 33516, Egypt; 10 School of Biotechnology, Badr University in Cairo, Cairo 11829, Egypt

**Keywords:** *Sophora*, *Glycyrrhiza glabra*, wound healing, UPLC/MS, flavonoids, phenolics, antioxidant, molecular docking

## Abstract

*Glycyrrhiza glabra* and *Sophora japonica* (Fabaceae) are well-known medicinal plants with valuable secondary metabolites and pharmacological properties. The flavonoid-rich fractions of *G. glabra* roots and *S. japonica* leaves were prepared using Diaion column chromatography, and the confirmation of flavonoid richness was confirmed using UPLC-ESI-MS profiling and total phenolics and flavonoids assays. UPLC-ESI-MS profiling of the flavonoid-rich fraction of *G. glabra* roots and *S. japonica* leaves resulted in the tentative identification of 32 and 23 compounds, respectively. Additionally, the wound healing potential of topical preparations of each fraction, individually and in combination (1:1) ointment and gel preparations, were investigated in vivo, supported by histopathological examinations and biomarker evaluations, as well as molecular docking studies for the major constituents. The topical application of *G. glabra* ointment and gel, *S. japonica* ointment and gel and combination preparations significantly increase the wound healing rate and the reduction of oxidative stress in the wound area via MDA reduction and the elevation of reduced GSH and SOD levels as compared to the wound and Nolaver^®^-treated groups. The molecular docking study revealed that that major compounds in *G. glabra* and *S. japonica* can efficiently bind to the active sites of three proteins related to wound healing: glycogen synthase kinase 3-*β* (GSK3-*β*), matrix metalloproteinases-8 (MMP-8) and nitric oxide synthase (iNOS). Consequently, *G. glabra* roots and *S. japonica* leaves may be a rich source of bioactive metabolites with antioxidant, anti-inflammatory and wound healing properties.

## 1. Introduction

Wound healing is a dynamic complicated process continuously presenting a clinical challenge. Pathologically, wound healing comprises four phases, namely, haemostasis, inflammation, proliferation and remodelling, respectively [1,2]. Many pharmaceutical preparations had been developed to maintain the healing process, including ointments, gels and wound dressings, in addition to surgical graft transplantations [3]. Herbal extracts and/or products derived thereof have been the basis for many formulations ensuring effectiveness, availability and safety [3,4,5]. Historically, medicinal plants have been the major component in the traditional medical systems, including the Chinese, Indian and Egyptian traditional systems, where they had been utilized in the curing and alleviating of different ailments [6,7]. *Aloe vera* [8], *Calendula officinal* [9], curcumin [10] and essential oils represent prominent examples with great contributions in one or more stages of the process of wound healing [1,11]. The efficacy of the different medicinal plants in wound healing could be related to the different classes of the secondary metabolites biosynthesised by medicinal plants, including triterpenoids, sterols (anti-inflammatory), flavonoids, polyphenolics (antioxidants), alkaloids (antimicrobial, anti-inflammatory) and/or essential oils (antimicrobial, anti-inflammatory) [12,13,14,15,16,17,18,19,20]. 

The roots and rhizomes of liquorice (*Glycyrrhiza glabra* Fam. Fabaceae) [21] are well known for their traditional uses in expectorant, demulcent, antibacterial and antiulcer drugs [22,23]; the different biological activities of liquorice can be traced to its high yield biosynthesis of triterpenoids, saponins and flavonoids [21]. Additionally, *G. glabra* is well known for its economic, nutritional value and medicinal impact as it has different biological functions, as antioxidant, anti-inflammatory, antiviral, anti-carcinogenic and anti-atherogenic [21,24]. Recently, liquorice extract in combination with lavender essential oil was reported to have wound healing potential [25]. 

The leaves of *Sophora japonica* (Fabaceae), known as Japanese pagoda, have been traditionally used as a haemostatic, hypotensive, detoxifying and anti-inflammatory agent as well [26,27]. The genus Sophora is known for its variety of secondary metabolites and biological activities [28,29]. The major secondary metabolites include flavonoids, isoflavonoids, triterpenoids and alkaloids [26,27,30,31,32,33]. The leaves of *S. japonica* exert a wide range of biological effects, including anti-inflammatory, antibacterial, anti-osteoporotic, antioxidant and whitening properties [27]. In addition, *S. flavescens* was reported to have wound healing potential [34].

Based on the prospective findings about the utilization of medicinal plants as promising treatments for wound healing, the role of *G. glabra* and *S. japonica* in wound healing and the extension of our concern in the therapeutic potential of herbal products, we herein report the investigation of the wound healing effects of both drugs either separately or in combination in gel and ointment formulations, evaluating their potential as appealing contenders for thoughtful drug development, encompassing in vivo comparative investigation of the potential of both plants under study either separately or in combination for healing wounds. The correlation of the biological findings to the chemical constituents of both plants in terms of UPLC-MS profiling is followed by the correlation of the major metabolites with their potential in wound healing cascades through a molecular docking study and the determination of the total phenolics and flavonoids of both drugs. 

## 2. Results

### 2.1. Total Phenolics and Total Flavonoids Contents

**Figure 1:**
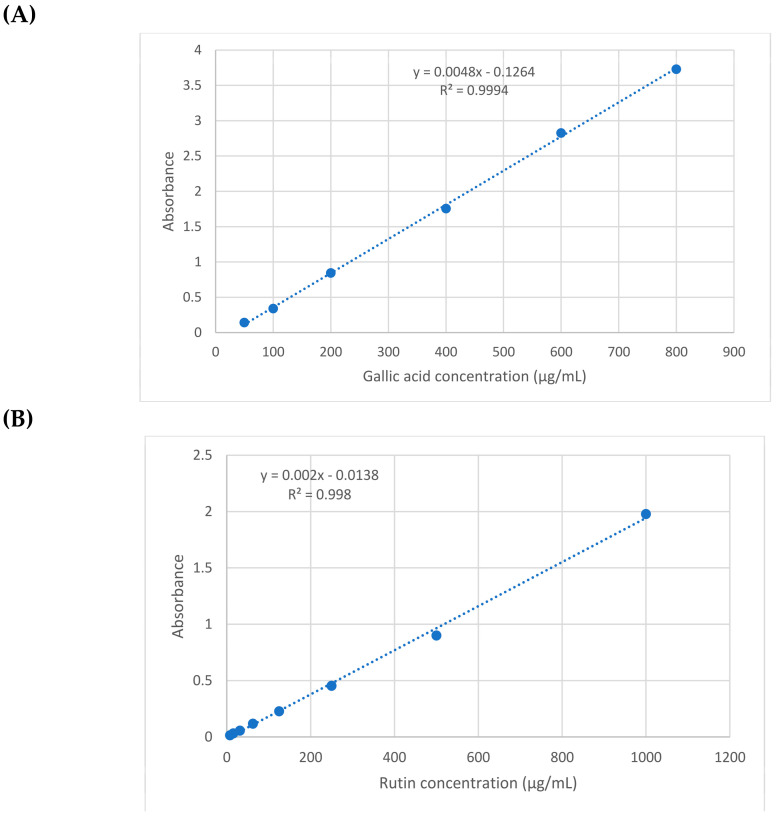
Calibration curve of (**A**) gallic acid and (**B**) quercetin.

The total phenolics (TPC) and flavonoids (TFC) contents in the *G. glabra* and *S. japonica* flavonoid-rich fractions were quantitatively determined [35,36]. Gallic acid and quercetin equivalents were used to assess phenolics and flavonoids contents. The TPC and TFC values were derived using the gallic acid calibration curve (*y* = 0.0048*x* − 0.1264 with R^2^ = 0.9994) and rutin (*y* = 0.002*x* − 0.0138 with R^2^ = 0.998), where *x* is the absorbance and *y* is gallic acid or rutin solution concentration (μg/mL), respectively (Figure 1). The presence of 71.608 ± 3.23 and 70.288 ± 1.94 μg/mg of GAE (gallic acid equivalent) per mg of *G. glabra* and *S. japonica* flavonoid-rich fractions extract were determined for the total phenolics (TPC). The existence of 46.99 ± 2.57 and 49.91 ± 2.36 μg QE/mg (quercetin equivalents) per mg of the *G. glabra* and *S. japonica* flavonoid-rich fractions extract were determined for the total flavonoids (TFC) (Table 1). The results established the presence of higher concentrations of the total phenolics in *G. glabra* and higher flavonoids contents in *S. japonica*. The results revealed that *S. japonica* is a rich source with phenolics as compared to the other species, *S. secundiflora* and *S. tomentosa*, which showed phenolics contents of 18.01 and 4.72 mg/g of GAE, respectively [28].

### 2.2. UPLC/MS Analysis of Glycyrrhiza glabra and Sophora japonica Flavonoid-Rich Fractions

Tentative metabolite identification was accomplished by extensive comparison of the UPLC-MS data from both extracts and the reported data [21,28,31,37,38,39], as well as online databases.

**Figure 2:**
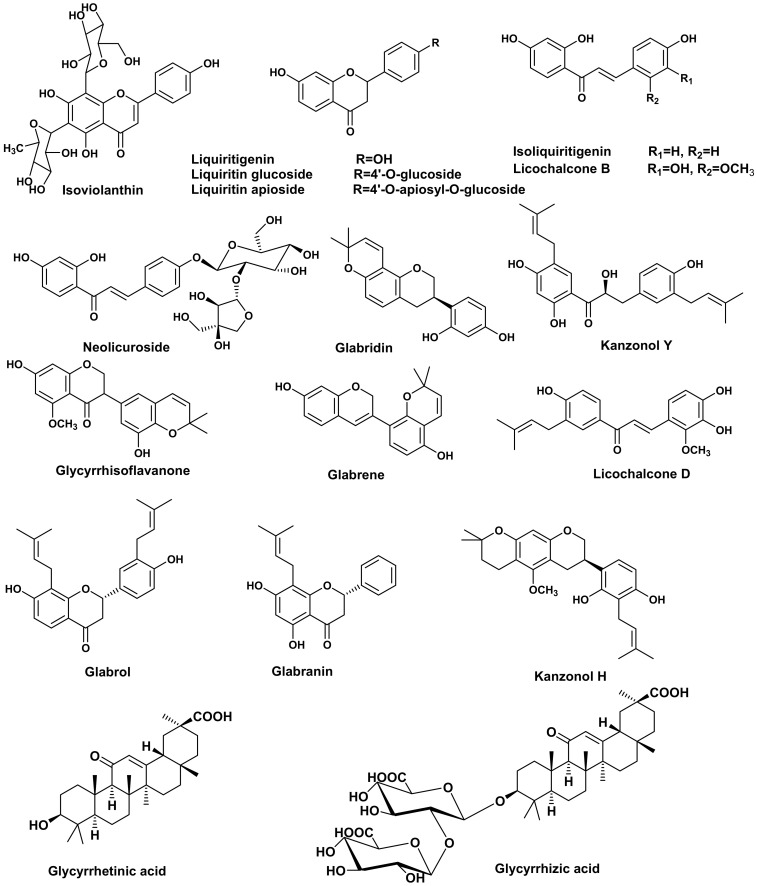
Major compounds identified in *G. glabra* flavonoid-rich fraction using UPLC-ESI-MS.

It is worth highlighting that polyphenolic components were the major constituents in both *G. glabra* and *S. japonica* flavonoid-rich fractions, with flavonoids and isoflavonoids being the most abundant classes. As demonstrated in (Table 2), a total of 32 compounds were tentatively identified in *G. glabra*, including 12 flavonoids, 8 chalcones, 5 triterpenoids, 5 isoflavonoids, 1 coumarin and 1 fatty acid. The mass ion peaks at *m*/*z* 577.15, 549.20, 417.25, 695.25 and 692.20, corresponding to the suggested molecular formulas C_27_H_30_O_14_, C_26_H_30_O_13_, C_21_H_22_O_9_, C_35_H_36_O_15_ and C_35_H_35_NO_14_, respectively, fit the flavonoid glycosides isoviolanthin, liquiritin apioside, liquiritin, licorice glycoside D2/D1 and licorice glycoside E. Aglycones with mass ion peaks at *m*/*z* 255.10, 257.10, 323.20, 407.20, 391.25, 339.10, 355.20, 323.20, 371.20, 323.20, 439.10 and 423.15, corresponding to the molecular formulas C_15_H_12_O_4_, C_15_H_12_O_4_, C_20_H_20_O_4_, C_25_H_28_O_5_, C_25_H_28_O_4_, C_20_H_20_O_5_, C_25_H_27_O_4,_ C_20_H_20_O_4_, C_21_H_20_O_6_, C_20_H_18_O_4_, C_27_H_34_O_5_ and C_26_H_32_O_5_, respectively, suggested flavonoid and isoflavonoids that were tentatively identified as liquiritigenin, 5,7-dihydroxyflavanone, glabranin, 3-hydroxyglabrol, glabrol, licoflavanone and isolicoflavonol, glabridin, glycyrrhisoflavanone, glabrene, licorisoflavan A, kanzonol H, respectively. Moreover, chalcones and chalcone glycosides were identified as licochalcone B, isoliquiritigenin, licochalcone D, licochalcone A, neolicuroside, echinatin, kanzonol Y and isoliquiritin, with mass ion peaks at *m*/*z* 287.15, 255.10, 353.20, 339.20, 549.20, 269.10, 409.20 and 417.15, respectively, correspond to the molecular formulas C_16_H_14_O_5_, C_15_H_12_O_4_, C_21_H_22_O_5_, C_21_H_22_O_4_, C_26_H_30_O_13_, C_16_H_14_O_4,_ C_25_H_30_O_5_ and C_21_H_22_O_9_, respectively. Additionally, the molecular ion mass peaks at *m*/*z* 837.40, 983.45, 821.40, 821.35 and 469.20, for the predicted molecular formulas C_42_H_62_O_17_, C_48_H_72_O_21_, C_42_H_62_O_16_, C_42_H_62_O_16_ and C_30_H_46_O_4_, gave hits of the triterpenes, licorice saponin G2, licorice saponin A3, licorice saponin K2/H2, glycyrrhizic acid and glycyrrhetinic acid, respectively, which were previously isolated from *G. glabra* [21]. Noteworthy is the presence of liquiritin apioside, neolicuroside, licorice saponin K2/H2, isoliquiritigenin, glycyrrhizic acid, glabridin, kanzonol Y, glabrol and glycyrrhetinic acid as the major constituents of the *G. glabra* flavonoid-rich fraction (Figure 2).

**Figure 3:**
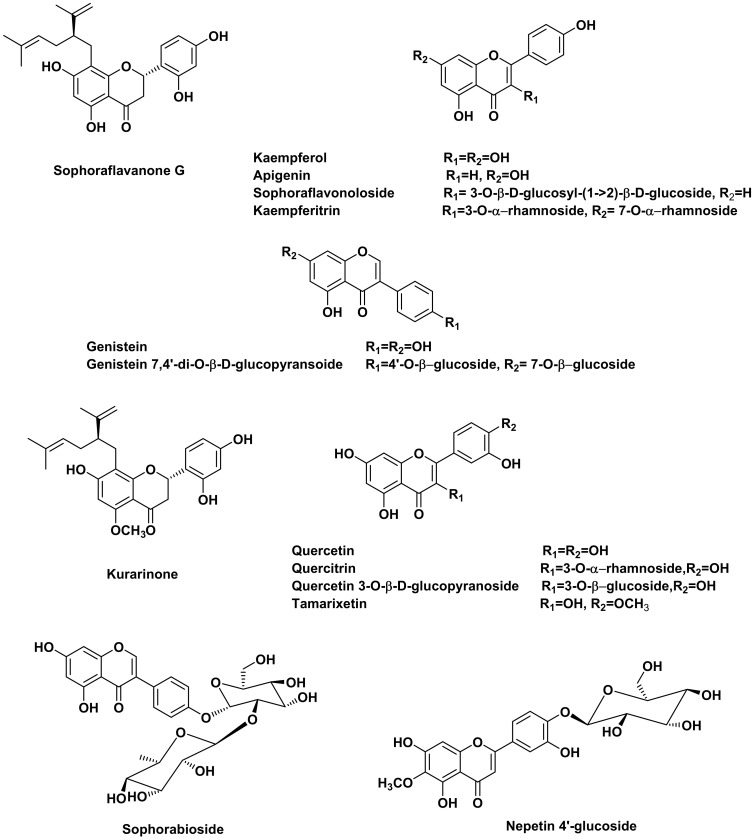
Major compounds identified in *S. japonica* flavonoid-rich fraction using UPLC-ESI-MS.

Regarding (Table 3), a total of 23 metabolites were tentatively identified in *S. japonica*, including 16 flavonoids, 4 isoflavonoids, 1 coumestan, 1 flavonostilben and 1 phenylpropanoid. The ion mass peaks at *m*/*z* 447.10, 267.50 and 901.25 [M − H]^−^ for the predicted molecular formulas C_21_H_20_O_11_, C_15_H_10_O_5_ and C_39_H_50_O_24_, respectively, gave hits of the quercitrin, apigenin and kaempferol 3-O-*α*-l-rhamnopyranosyl(1→6)-*ꞵ*-d-glucopyranosyl(1→2)-*ꞵ*-D-glucopyranoside-7-O-*α*-l-rhamnopyranoside, which were isolated from *S. japonica* [31,33]. The ion mass peaks at *m*/*z* 609.20, 593.10 and 577.20 [M − H]^−^ for the suggested molecular formulas C_27_H_30_O_16_, C_27_H_30_O_15_ and C_27_H_30_O_14_, respectively, correspond to sophoraflavonoloside, genistein 7,4′-di-O-*ꞵ*-D-glucopyransoide and sophorabioside, which were isolated from *S. japonica* seeds [43]. Two ion peaks values at *m*/*z* 755.25 and 739.20 [M − H]^−^ with the molecular formulas C_33_H_40_O_20_ and C_33_H_39_O_19_, respectively, were tentatively identified as genistein 7-*O*-*ꞵ*-D-glucopyranoside-4′-*O*-[(*ꞵ*-D-glucopyranosyl)- (1→2)- *β*-D-glucopyranoside] and genistein 7-O-*ꞵ*-D-glucopyranoside-4′-O- [(*α* -L-rhamnopyranosyl) -(1→2)- *ꞵ*-D-glucopyranoside], which were previously isolated from *S. japonica* [44]. The ion mass peaks at *m*/*z* 463.25 and 461.15 [M − H]^−^ corresponding to the molecular formulas C_21_H_19_O_12_ and C_22_H_22_O_11_, respectively, were detected and dereplicated as quercetin 3-O-*β*-D-glucopyranoside and paratensein-7-*O*-glucoside, which were isolated from the small branches and stem bark of *S. japonica,* respectively [37,38]. Moreover, flavonoid and isoflavonoid aglycones with their ion mass peaks values at *m*/*z* 301.20, 269.45, 283.10 [M − H]^−^ and 317.25 [M + H]^+^ corresponding to C_15_H_10_O_7_, C_15_H_10_O_5_, C_15_H_10_O_6_ and C_16_H_12_O_7_, respectively, gave hits of quercetin, genistein, kaempferol and tamarixetin, respectively, which were previously isolated from *S. japonica* [45,46]. In addition, two main peaks with *m*/*z* values 425.20 [M − H]^−^ and 439.50 [M + H]^+^, corresponding to the molecular formulas C_25_H_28_O_6_ and C_26_H_30_O_6_*,* respectively, hit sophoraflavanone G and kurarinone, which were previously isolated from *S. flavescens* [47]. Noteworthy is that sophoraflavanone G, sophoraflavonoloside, genistein 7,4′-di-O-*β*-D-glucopyransoide, kurarinone, genistein, kaempferol and tamarixetin are the most abundant constituents in the *S. japonica* flavonoid-rich fraction (Figure 3).

### 2.3. In Vivo Wound Healing Experiment

#### 2.3.1. Effect of Topical Application of Different Treatments on Wound Contraction

**Figure 4:**
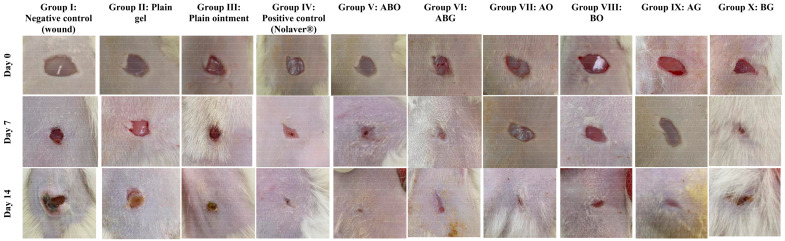
Representative images demonstrate the excised wounds during the healing process in different rats group after applying various treatments topically on days 0, 7 and 14. ABO: Ointment of *G. glabra* and *S. japonica* combination (1:1), ABG: Gel of *G. glabra* and *S. japonica* combination (1:1), AO: Ointment of *G. glabra,* BO: Ointment of *S. japonica*, AG: Gel of *G. glabra*, BG: Gel of *S. japonica*.

**Figure 5:**
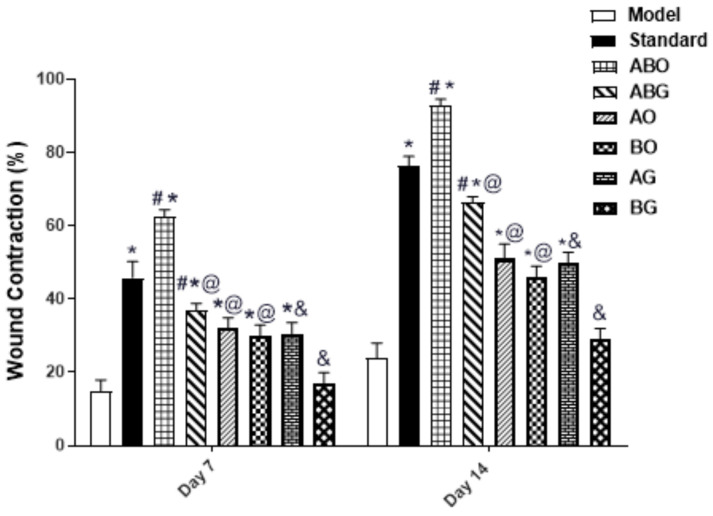
Effects of various treatments on the rate of wound healing at various days in rats (wound contraction, %). Values are expressed as mean ± SD (*n* = 6). The symbols *, #, @, & indicate statistical significance at *p* < 0.05, symbol * as compared to the model, symbol # as compared to the standard, symbol @ as compared to the (ABO) ointment of the *G. glabra* and *S. japonica* combination (1:1) and symbol & as compared to the (ABG) gel of the *G. glabra* and *S. japonica* combination (1:1), using a two-way ANOVA followed by the Bonferroni post hoc test; *p* < 0.05.

The percentage reduction in wound area was calculated to determine the extent of the wound contraction [51]. As illustrated in (Figure 4 and Figure 5), the wound contraction was significantly improved by the topical application of Nolaver, ABO, ABG, AO, BO and AG preparations, with a remarkable increase in Nolaver^®^, ABO, and ABG groups by 3-,4- and 2.8-fold, respectively, in comparison to the wound model group on day 7. Furthermore, only the percentage of wound contraction in the ABO group was dramatically higher than that in the Nolaver group, by 36%. By the same mean, on day 14 of the experiment, the topical application of Nolaver, ABO and ABG significantly accelerated wound healing by 3-, 3.8- and 3-fold in contrast to the wound model group. As expected, only the ABO group showed an outstanding effect compared to the rest of treatment. Noteworthy is that the *G. glabra* and *S. japonica* flavonoid-rich fractions combination, either ointment or gel (ABO and ABG), significantly improved wound contraction compared to their individual constituents (AO, BO) and (AG, BG), respectively.

In this work, we selected two different formulations as delivery systems for the extracts of the investigated medical plants: ointment and hydrogel. The selection of the post-mentioned formulations was based on two factors. The first factor is the formulation nature (hydrophilicity/hydrophobicity), and the second factor is the native wound healing capacity of the plain formulation itself. Regarding the nature of the formulation, it greatly affects the release behaviour of the drug. Hydrophilic drugs are better to be incorporated into formulations with lipophilic characters in order to enhance drug partitioning between the formulation and the applied tissues. On the contrary, the formulation that achieves the complete solubilization of the drug will result in low drug diffusion towards the applied skin. Therefore, the simple ointment as a hydrophobic delivery system was investigated to deliver the alcoholic and hydroalcoholic extracts of the medical plants (more hydrophilic). For the fulfilment of the second factor, hydrogels are thought to be an effective carrier for the topical delivery of various drugs intended for wound healing. The high-water content supplies the hydration required for healing process. The in vivo experiment results revealed that ointment formulations significantly accelerated wound healing over hydrogels for both the single and combination preparations, as shown in (Figure 4). This observation endorses the importance of the proper selection of the base that achieves optimum partitioning and diffusion of the drug.

#### 2.3.2. Histopathology

**Figure 6:**
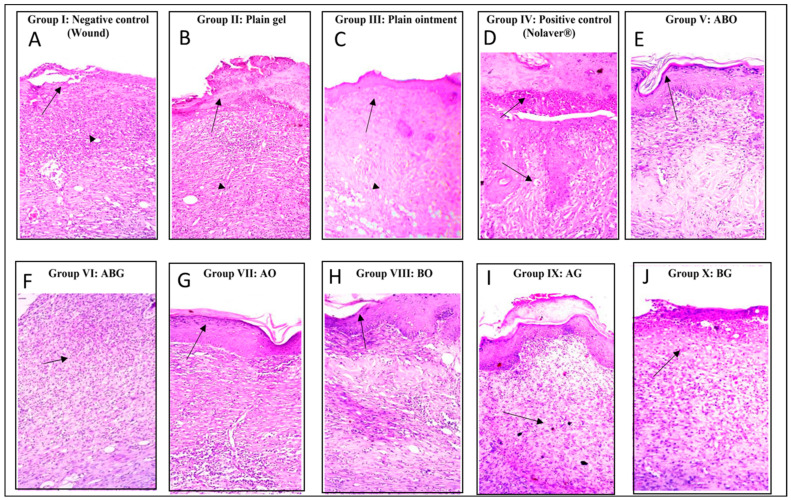
Histopathological view of wound healing and epidermal/dermal remodelling in the groups administered different treatments on day 14. (**A**) Thickening of epidermis at its cut edges by necrotic tissues with massive inflammatory cell infiltration, mainly neutrophils **arrow.** (**B**) Thickening of epidermis by inflammatory cells and necrotic tissues with mild neo-angiogenesis and new vessel formation in dermal layer **arrow**. (**C**) Thickening of epidermis by inflammatory cells and necrotic tissues with mild neo-angiogenesis and new vessel formation in dermal layer **arrow**. (**D**) Granulation tissue consisted mainly of fibroblasts and migration of epithelial cells (< 50%) **arrow**. (**E**) Thickening and migration of epithelial cells (< 50%), newly created granulation tissue and keratinization epithelial layer **arrow**. (**F**) Massive inflammatory cell infiltration, mainly neutrophils and non-organized collagen **arrow**. (**G**) Migration of epithelial cells (≥ 50%) and keratinization epithelial layer **arrow**. (**H**) Migration of epithelial cells (≥ 50%) and keratinization epithelial layer **arrow**. (**I**) Newly created granulation tissue rich on inflammatory cell cells, mainly neutrophils arrow. (**J**) Massive inflammatory cell infiltration, mainly neutrophils and non-organized collagen **arrow**. ((**H**,**E**)X200).

Fourteen days post-treatment, as shown in (Figure 6) and scored in (Table 4), the examination of the negative control slides under a microscope (Group I) and both plain treatments (Group II and III) samples revealed slow wound healing with a significant presence of ulcers, scabs, necrotic tissues and infiltrating inflammatory cells, mainly neutrophils (arrow). There was an abundance of inflammatory cells in the highly cellular granulation tissue and numerous activated fibroblasts in the dermis, as well as newly developed blood vessels. However, the positive control (Nolaver^®^) (Group IV) samples showed a rapid recovery from the wound, with the epidermis completely re-epithelialized (arrows) with very moderate vascular alterations in basal keratinocytes and more mature collagen fibres formed. In addition, the granulation tissue containing numerous fibroblasts shrank. In Group V: Ointment of *G. glabra* and *S. japonica* combination (1:1) (ABO), the epidermal layer completely re-epithelialized. There is a large region of dermal granulation tissue that was highly cellular and less fibrous, with an abundance of tiny blood capillaries. The sub-epithelial haemorrhages were localised in clusters. Similarly, Ointment of *G. glabra* (AO) Group VII revealed a wound that was showing signs of healing, including new collagen and a slight presence of inflammatory cells. Group IX: Gel of *G. glabra* (AG), Group X: Gel of *S. japonica* (BG) and Group VIII: Ointment of *S. japonica* (BO) showed an incomplete wound healing and thick epidermis with a marked presence of inflammatory cells, mainly neutrophils. In Group VI: Gel of *G. glabra* and *S. japonica* combination (1:1) (ABG), there showed incomplete wound healing, with a fewer number of inflammatory cells.

#### 2.3.3. Estimation of Thiobarbituric Acid Reactive Substances (TBARS) Level Expressed as Malondialdehyde (MDA)

**Figure 7:**
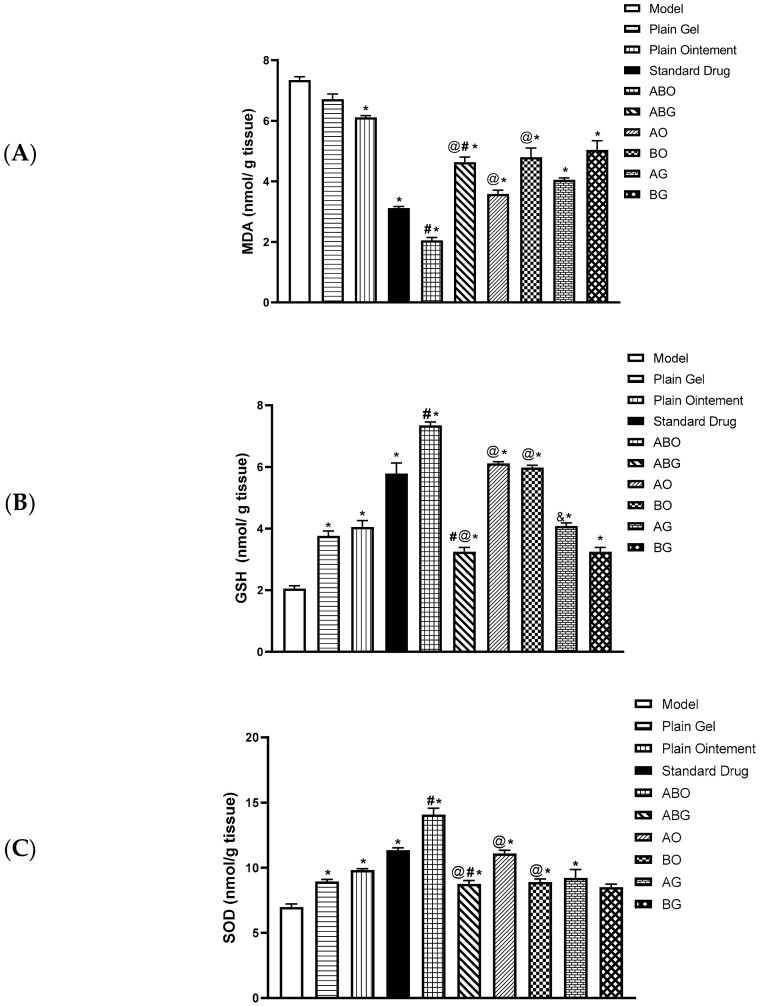
Thiobarbituric acid reactive substances (TBARS) level expressed as malondialdehyde (MDA); (**A**) reduced glutathione (GSH) (**B**) and superoxide dismutase activity (SOD) (**C**) in the wound tissues. (%). Values are expressed as mean ± SD (*n* = 6). *, #, @, & indicate *p* < 0.05, compared to Groups I, IV (Standard), V (ABO) and VI (ABG), respectively, using a one-way ANOVA followed by Tukey’s post hoc test; *p* < 0.05.

The wound group showed an eminent increment of the MDA level, an indicator of lipid peroxidation. The topical application of Nolaver^®^, *G. glabra* ointment and gel (AO and AG)*, S. japonica* ointment and gel (BO, BG) and the combination preparations (ABO, ABG) significantly attenuated the lipid peroxidation with a remarkable decrease in MDA levels in the positive control group and the ABO groups by 2.4- and 3.7-fold, respectively, compared to the model group. Moreover, the combination ointment preparation (ABO) only showed a significant decrement in MDA by 1.5-fold in comparison to the positive control group. However, the rest of the treatments revealed a statistically significant elevation in MDA in comparison to the ABO group. When compared to the single ointment preparation of *G. glabra* and *S. japonica* (AO and BO), the (ABO) preparation significantly reduced MDA. However, the MDA level in the single gel formulation of *G. glabra* and *S. japonica* (AG and BG) groups did not differ significantly when compared with the combination gel preparation (ABG) group (Figure 7).

#### 2.3.4. Estimation of Reduced Glutathione GSH and SOD Activity in the Wound Tissues

The wound injury in the model group resulted in a remarkable decrease in glutathione (GSH) level and superoxide dismutase (SOD) activity, two key antioxidant tissue components. The topical application of Nolaver^®^, *G. glabra* ointment and gel (AO and AG)*, S. japonica* ointment (BO) and combination preparations significantly increased the GSH level and SOD activity compared to the model group. The combination ointment preparation (ABO) significantly increased the GSH levels (by 3.7- and 1.3-fold, respectively, in comparison to the model and positive control groups) and restored SOD activity (by 2- and 1.3-fold, respectively, in comparison to the model and positive control groups). Interestingly, the combination ointment preparation (ABO) significantly increased both GSH levels and SOD activity as compared to single ointment preparation of *G. glabra* and *S. japonica* (AO and BO). Except for GSH in the single gel preparation of *G. glabra* (AG) group, no statistically significant differences regarding GSH level or SOD activity were observed between the single gel preparations of *G. glabra* and *S. japonica* (AG and BG) groups versus the combination gel preparation (ABG) group (Figure 7).

#### 2.3.5. Evaluation of CDI for the Combination

To study the effects of the interaction for the combination in gel and ointment formulations, CDI was estimated for the wound contraction percent besides the influence on the MDA, GSH and SOD levels. The results are represented in (Table 5), displaying synergistic effects. The CDI determination is a helpful approach for determining the type of therapeutic interactions. The current study examined the consequences of wound healing of *G. glabra* and *S. japonica* flavonoid-rich fractions at a single concentration (10%) and in combined formulations of ointment and gel. The CDI for the effect of the combination of G. glabra and S. japonica flavonoid-rich fractions in both formulation ointment and gel on all parameters investigated; wound contraction percent, MDA, GSH and SOD level was calculated to be synergistic.

In the current study, ointment and gel topical preparations prepared with either the flavonoid-rich fractions of *G. glabra, S. japonica* or a combination of two fractions were assessed for their wound healing capacity. To shed light on how the formulation’s components interact synergistically, each fraction was assessed separately for its wound healing efficacy. Wound healing efficacy was investigated through the antioxidant markers, viz., MDA, reduced GSH and SOD levels.

Different extracts of *Glycyrrhiza glabra* revealed broad dermatological applications, including treating a variety of skin conditions and infections [52]. The primary antioxidative and anti-inflammatory properties of *G. glabra* are the basis for the reported skin benefits [24,39,53]. Different extracts of *G. glabra* are recently embedded in variable skin products due to its richness with flavonoids and its two primary active ingredients, glycyrrhizin and glycyrrhetinic acid, which are powerful inhibitors of cortisol metabolism [24,52,54]. Saeedi et al. (2003) revealed the use of liquorice as an effective treatment for skin dermatitis [55]. Several reports revealed the important contribution of major constituents of *G. glabra*, glycyrrhetinic acid, glycyrrhizin, glabridin, isoliquiritigenin, licochalcone A and liquiritin, in the management of skin conditions, owing to their notable antimicrobial, antioxidant and anti-inflammatory effects [56,57,58,59,60,61,62]. In addition, the flavonoids of *S. japonica* are reported for their antioxidant, antimicrobial and anti-inflammatory properties [63], besides their role in skin conditions as contact dermatitis [14,64]. It has been shown that sophoraflavanone G has various activities, including being antimicrobial, antioxidant and anti-inflammatory, along with a limited cytotoxicity, valuable for wound healing [65].

In accordance with previous investigations, the current study revealed that the groups treated with a combination of *G. glabra* and *S. japonica* (1:1) in ointment formulation interestingly showed that improved wound contraction and oxidative stress markers (as observed by decreased lipid peroxidation and higher GSH and SOD levels), as well as enhanced re-epithelialization as compared to the negative control group in the histopathological examination. The antioxidant and wound healing potential observed in the current study are significantly influenced by the abundance of various flavonoids in both fractions of *G. glabra* and *S. japonica*.

### 2.4. Molecular Docking

This section investigated the various mechanisms by which the main compounds mentioned above exert biological effects. Consequently, using the following IDs: 3F88, 5H8X and 3N2R for glycogen synthase kinase 3-*β* (GSK3-*β*), matrix metalloproteinases-8 (MMP-8) and nitric oxide synthase (iNOS), respectively, their 3D structures were obtained from the protein data bank. The RMSD values between the co-crystalized and the retrieved docking poses were 0.78, 1.12 and 0.85 Å, for 3F88, 5H8X and 3N2R, respectively indicating valid docking protocol (see Supplementary Information). Following that, the fifteen major compounds were docked in the vicinity of the active sites of the three enzymes. It was obvious that after docking with the three targets, all compounds achieved acceptable binding scores (Table 6).

#### 2.4.1. Docking of *Glycyrrhiza glabra* Major Compounds

**Figure 8:**
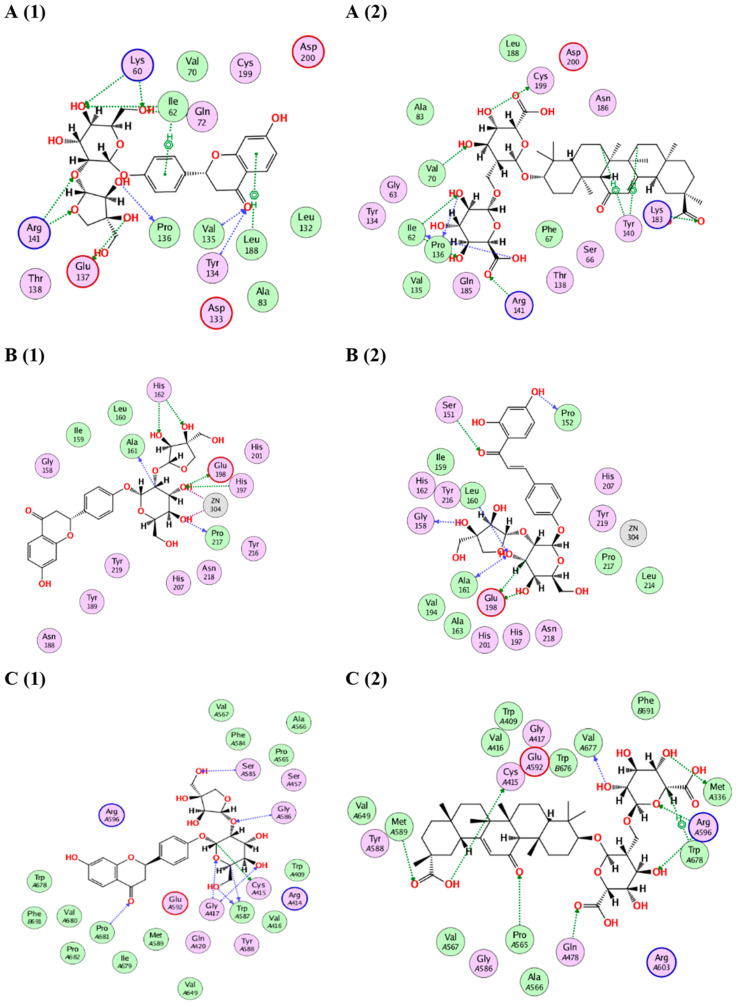
(**A**) The 2D binding modes of liquiritin apioside (**A1**) and glycyrrhizic acid (**A2**) to the active binding sites of GSK3-*β*. (**B**) The 2D binding modes of liquiritin apioside (**B1**) and neolicuroside (**B2**) to the active binding sites of MMP-8. (**C**) The 2D binding modes of liquiritin apioside (**C1**) and glycyrrhizic acid (**C2**) to the active binding sites of iNOS.

The major identified compounds in *Glycyrrhiza glabra* (liquiritin apioside, neolicuroside, isoliquiritigenin, glycyrrhizic acid, glabridin, kanzonol Y, glabrol and glycyrrhetinic acid) exerted synergetic effects as indicated by the acceptable docking scores of all the identified compounds (Table 6). In the docking of GSK3-*β*, liquiritin apioside and glycyrrhizic acid obtained the highest docking scores of −14.1 and −15.2 Kcal/Mol, respectively. As shown in (Figure 8(A1, A2)), liquiritin apioside interacted with Val135, Tyr134, Pro136, Glu137, Arg141, Lys60, Ile62 and Leu188, and glycyrrhizic acid interacted with Val70, Lys183, Tyr140, Pro136, Arg141, Ile62 and Cys199. In the docking of matrix metalloproteinases-8 (MMP-8), liquiritin apioside and neolicuroside achieved the best docking scores of −12.4 and −15.4 Kcal/Mol, respectively. As depicted in (Figure 8(B1, B2)), liquiritin apioside bound to MMP-8 through interactions with Ala161, His162, His197, Glu198, Zn304 and Pro217, while neolicuroside interacted with Ser151, Pro152, Gly158, Leu160, Ala161 and Glu198. In the docking of nitric oxide reductase (iNOS), liquiritin apioside and glycyrrhizic acid achieved the best docking scores of −14.5 and −18.2 Kcal/Mol, respectively. Inspecting (Figure 8(C1, C2)), liquiritin apioside was able to interact with the residues of iNOS through binding with Cys415, Gly417, Ser585, Gly586, Trp587 and Pro681, and glycyrrhizic acid interacted with Met336, Cys415, Gln478, Pro565, Met589, Arg596, Val677 and Trp678. In conclusion, the docking results validated and confirmed the biological findings, leading to a synergistic impact for all the major *G. glabra* extract constituents.

#### 2.4.2. Docking of *Sophora japonica* Major Compounds

**Figure 9:**
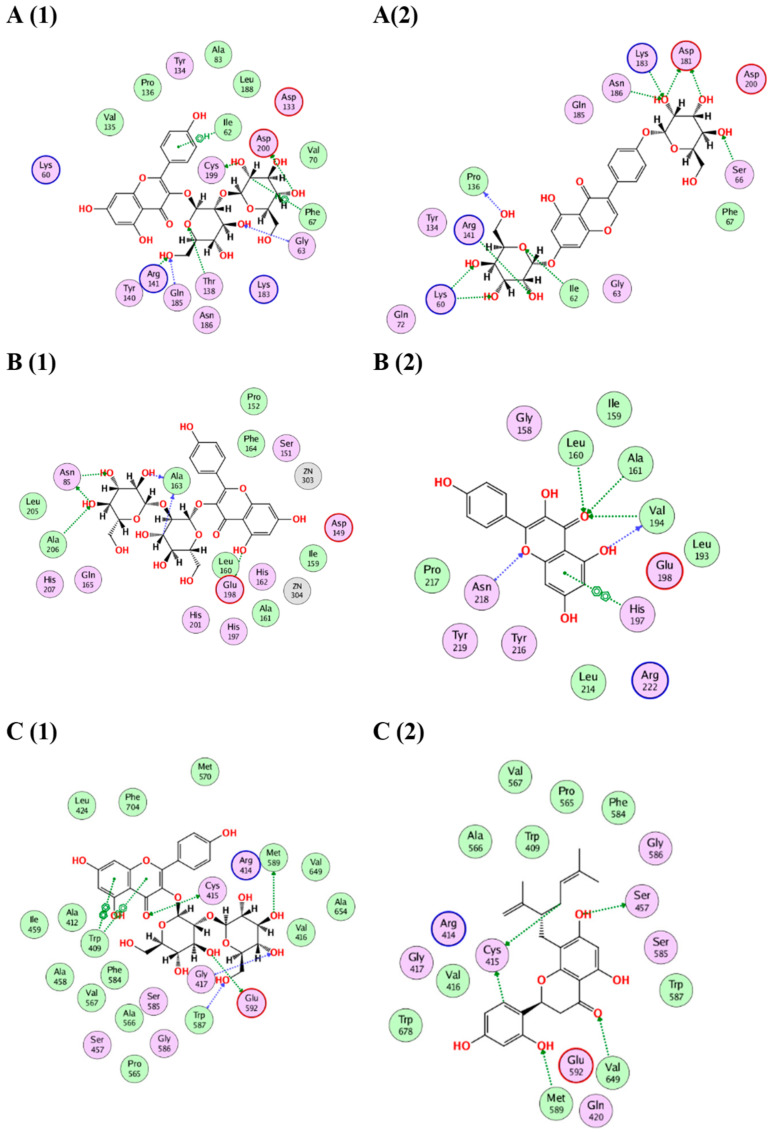
(**A**) The 2D binding modes of sophoraflavonoloside (**A1**) and genistein 7,4′-di-O-*ꞵ*-D-glucopyransoide (**A2**) to the active binding sites of GSK3-*β*. (**B**) The 2D binding modes of sophoraflavonoloside (**B1**) and kaempferol (**B2**) to the active binding sites of MMP-8. (**C**) The 2D binding modes of sophoraflavonoloside (**C1**) and sophoraflavanone G (**C2**) to the active binding sites of iNOS.

The isolated major compounds (sophoraflavanone G, sophoraflavonoloside, genistein 7,4′-di-O-*ꞵ*-D-glucopyranoside, kurarinone, genistein, kaempferol and tamarixetin) exerted synergetic effects as indicated by the acceptable docking scores of all the identified compounds (Table 6). In the docking of GSK3-*β*, sophoraflavonoloside and genistein 7,4′-di-O-*ꞵ*-D-glucopyranoside achieved the best docking scores of −14.3 and −16.9 Kcal/Mol, respectively. As shown in (Figure 9(A1, A2)), sophoraflavonoloside interacted with Ile62, Gly63, Phe67, Thr138, Arg141, Gln185, Cys199 and Asp200, and genistein 7,4′-di- O-*ꞵ*-D-glucopyranoside interacted with Lys60, Ile62, Ser66, Pro136, Arg141, Asp181, Lys183 and Asn186. In the docking of matrix metalloproteinases-8 (MMP-8), sophoraflavonoloside and kaempferol achieved the best docking scores of −13.7 and −13.4 Kcal/Mol, respectively. As depicted from (Figure 9(B1, B2)), kaempferol interacted with Leu160, Ala161, Val194, His197 and Asn218. Sophoraflavonoloside bound to MMP-8 through interactions with Asn85, Ala163, Glu198 and Ala206. In the docking of nitric oxide reductase (iNOS), sophoraflavonoloside and sophoraflavanone G achieved the best docking scores of −16.1 and −14.6 Kcal/Mol, respectively. Inspecting (Figure 9(C1, C2)), sophoraflavonoloside was able to interact with the residues of iNOS through binding with Trp409, Cys415, Gly417, Trp587, Met589 and Glu592, while sophoraflavanone G interacted with Cys415, Ser457, Met589 and Val649. In conclusion, the docking results validated and confirmed the biological findings, leading to a synergistic impact for all the major *S. japonica* extract constituents.

### 2.5. Pharmacokinetic Profiling

**Figure 10:**
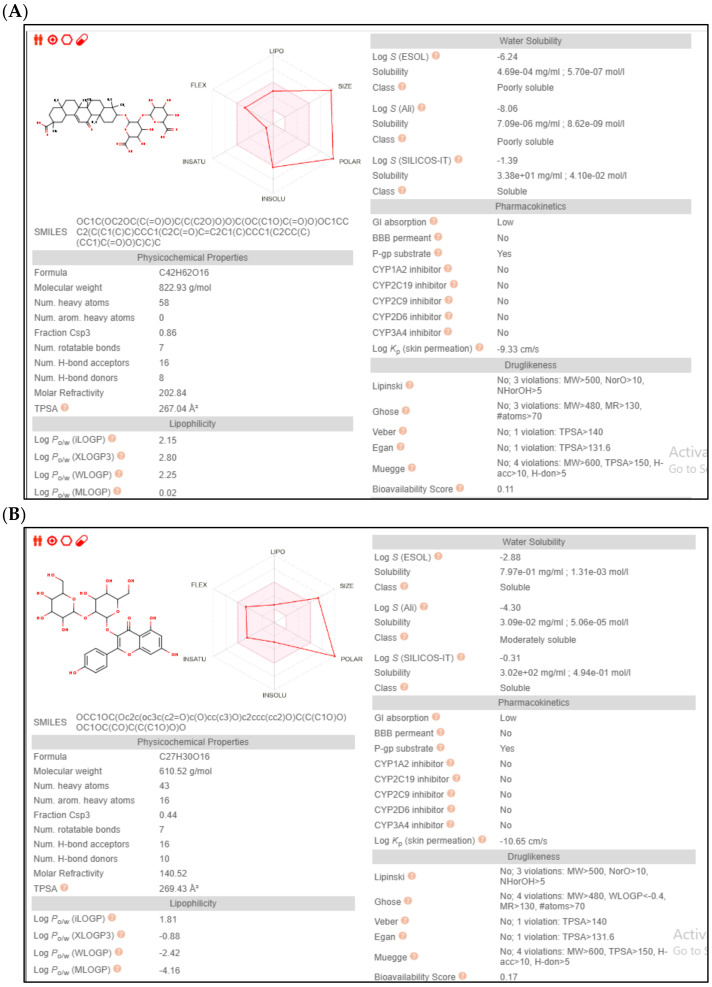
The pharmacokinetic profiling of compounds (**A**) glycyrrhizic acid and (**B**) sophoraflavonoloside.

It is well established that drug candidates should have both acceptable pharmacological and pharmacokinetic profiles. Accordingly, the ADME profile of glycyrrhizic acid and sophoraflavonoloside were calculated using SWISS ADME. In general, both compounds showed a low degree of absorption from the gastrointestinal tract (GIT). This is probably attributed to the high polarity of both compounds that violate the required physicochemical properties for optimum absorption. As demonstrated by the properties radar chart, both the compounds had the desired values of all the properties (size, polarity, lipophilicity, flexibility, solubility and saturation) with exception for the size and polarity (Figure 10). Moreover, it is very important to get insights in the metabolic behaviour of both the compounds. Both compounds were found to have no interactions with various isoforms of cytochrome enzymes, including CYP1A2, CYP2C19, CYP2C9, CYP2D6 and CYP3A4; thus they could be used safely with other drugs with no need for dose adjustment. A worthy note is that both compounds had no violation of any of the drug-likeness rules (Lipinski, Viber, Muegge, Ghose, Veber and Egan) making them excellent drug candidates for future optimization. Finally, both compounds have no records in pan interference assays (PAINS), giving rise to their potential high safety margin.

## 3. Materials and Methods

### 3.1. Plant Material Extraction, and Fractionation

The roots of *G. glabra* were purchased from a local market in Egypt in November 2020. The leaves of *S. japonica* were obtained from the El-Orman Botanical Garden, Giza, Egypt, in December 2020. Both plants had their authenticity verified by taxonomy specialist engineer, Therease Labib, El-Orman Botanical Garden, Giza, Egypt. Plant material voucher specimens, under code BUC-PHG-GG-1 for *G. glabra* and BUC-PHG-SJ-2 for *S. japonica*, were placed at the Pharmacognosy Department, Faculty of Pharmacy, Badr University in Cairo.

The air-dried pulverized leaves of *S. japonica* (250 gm) and the roots of *G. glabra* (500 gm) were separately macerated in 70% methanol (3 × 500 mL) and (3 × 1 L) for *S. japonica* and *G. glabra*, respectively, followed by filtration. The filtrate was completely evaporated in vacuo at a low temperature (45 °C), using a rotary evaporator (Hei-VAP Value, Heidolph) to produce dry residue (59 g; 23.6% *w*/*w*) and (83.6 g; 16.72% *w*/*w*), respectively. The extraction yield was determined by the equation: [total weight of dried residue/total weight of fresh plant] × 100 [66]. Then, each extract (50 g) was fractionated separately on Diaion HP-20 (SUPLECO, North Harrison Road, Bellefonte, PA, USA) using a gradient concentration of methanol/water to obtain four main fractions for each plant: 100% water, 25% methanol, 75% methanol and 100% methanol. The 75% methanol fraction is the flavonoid-rich fraction that produces a yellow colour with NH_3_ vapour and a green colour with FeCl_3_ [67]. The flavonoid-rich fraction (20 g) for *G. glabra* and (13 g) for *S. japonica* were kept in tightly sealed containers for further biological and phytochemical investigations.

### 3.2. Total Phenolics and Total Flavonoids

The total phenolic content of the *G. glabra* and *S. japonica* flavonoid-rich fraction was determined using the Folin–Ciocalteu method, as described by Attard [35]. Briefly, we started with mixing 10 μL of sample/standard with 100 μL of the Folin–Ciocalteu reagent (diluted 1:10) in a 96-well microplate. Afterwards, 80 μL of 1M Na_2_CO_3_ was added and incubated at room temperature (25 °C) for 20 min in the dark. Following the incubation period, the blue complex colour that resulted was detected at 630 nm. Data represented as means ± SD and the gallic acid % was estimated using a pre-established standard calibration curve. The total phenolic content was expressed in μg of the gallic acid equivalents/mg extract.

The total flavonoids content determined using the aluminium chloride method, as described by Kiranmai [36], with some modifications was conducted on microplates. In brief, 15 μL of the sample/standard was placed in a 96-well microplate, then, 175 μL of methanol was added, followed by 30 μL of 1.25% AlCl_3_. At the end, 30 μL of 0.125 M C_2_H_3_NaO_2_ was added and incubated for 5 min. Following the incubation period, the yellow colour was measured at 420 nm. Data represented as means ± SD and with reference to a previously created standard calibration curve, the % was estimated as quercetin. The FluoStar Omega microplate reader was used to record the results.

### 3.3. UPLC-ESI-MS Analysis 

UPLC/MS analysis was performed at the Centre of Drug Discovery Research and Development, Department of Pharmacognosy, Faculty of Pharmacy, Ain Shams University, Egypt, using Waters^®^ TQD UPLC-MS with an ESI source using Waters^®^ Acquity UPLC RP-C18 column, (100 × 2 mm, ID), and a particle size of 1.7 μm, with an integrated pre-column. From 2% to 100% acetonitrile, a gradient of water and acetonitrile was applied, along with 0.1% formic acid. The flow rate was 1 or 0.5 mL/min and one run took 35 min. The MS was operated at −10 V for ESI-, a 240 °C source temperature and high purity N_2_ was used as a sheath and auxiliary gas at a flow rate of 80 and 40 (arbitrary units), respectively. The injection volume was 5 μL. The voltage of 4.48 kV was used as a spray voltage; 10.00 V was the tube lens, and 39.6 V was the capillary voltage. A full scan mode was adjusted in the mass range of 100–2000 *m*/*z*. The compounds were tentatively identified using MS data (in the negative and positive ionization mode) in comparison to previously known compounds from the genus and family. XcaliburTM 2.0.7 software was used for collecting data and analysis (Thermo Scientific, Karlsruhe, Germany) [68].

### 3.4. Preparation of Topical Extract Gel

An amount equivalent to 1.5% *w*/*w* of carbopol 940 was stirred for 60 min in distilled water containing 0.01% w/v benzalkonium chloride as a preservative. Propylene glycol (10% *w*/*w*) was then added to form a gel dispersion. Alcoholic and hydroalcoholic herbal extracts (equivalent to 10% *w*/*w*) were gradually added to the gel system while being constantly stirred. Finally, the gel was developed spontaneously by adding triethanolamine dropwise, and the pH of the preparation was adjusted to 7. Mixing continued until a transparent gel was obtained [69,70].

### 3.5. Preparation of Topical Extract Ointment

Simple ointments were made from the extracts of the plant materials under study. The ointment was prepared according to the British Pharmacopoeia [71] as follows (Table 7):

Reduced amounts of the ingredients, required to prepare 25 g of the ointment base, were combined, gently heated while being stirred to obtain homogeneity and then stirred continuously until the base cooled and congealed. For the preparation of medicated ointments, 10% *w*/*w* of the herbal extracts was added to the melted base of simple ointments. 

### 3.6. In Vivo Wound Healing Experiment

#### 3.6.1. Animals

Sixty adult male Wistar albino rats, weighing approximately 200–250 g, were obtained from the animal house at the Faculty of Pharmacy, Badr University in Cairo (Cairo, Egypt). They were kept in plastic cages in a standard laboratory environment (23 ± 1 °C, 40–60% humidity, 12 h light/dark cycles), fed standard rat pellet food and were allowed to drink water ad libitum. Before the study, the rats were adapted to their new environment for one week before the experiment. The Research Ethics Committee of the Faculty of Pharmacy at Badr University in Cairo approved the experimental procedures (PG-117-A), which followed the rules set by the US National Institutes of Health for the proper care and use of laboratory animals (NIH Publication No. 85-23, revised 2011).

#### 3.6.2. Wound Induction and Experimental Groups

To induce wounds in an animal model, each rat was anaesthetized with ketamine hydrochloride at a dose of (100 mg/kg i.p.) Then, the rat’s anterior dorsal side was shaved using a sterile surgical blade and a patch of skin was removed to create a full thickness excision wound of two cm^2^. The skin was checked for any irritation or scars [4].

On the following day, the rats were randomly assigned into ten groups of six rats each, as follows: 

Group I: Negative control (Wound)

Group II: Plain gel

Group III: Plain ointment

Group IV: Positive control (Nolaver^®^)

Group V: Ointment of *G. glabra* and *S. japonica* combination (1:1) (ABO).

Group VI: Gel of *G. glabra* and *S. japonica* combination (1:1) (ABG).

Group VII: Ointment of *G. glabra* (AO).

Group VIII: Ointment of *S. japonica* (BO).

Group IX: Gel of *G. glabra* (AG).

Group X: Gel of *S. japonica* (BG).

Throughout the experiment, the wounds were firstly cleaned with 0.9% saline solution and a thin layer of each formulation was applied and evenly distributed over the wound surface once daily for 14 consecutive days. Then animals were caged individually to prevent them from biting the wounds. The healing of the wounds was evaluated daily. On day 14, the last day of the study, the rats were euthanized by decapitation under anaesthesia, using thiopental (50 mg/kg), and the wound granulation tissues produced were removed for further investigation. Buffered formalin was used for H&E staining and histopathological examination, while a phosphate buffer solution was used for biochemical assessment [72].

#### 3.6.3. Wound Contraction Measurements

The wound contraction percentage was estimated using the procedures outlined in [73]. Rats were aligned on a workbench with the wound facing up to measure the entire wound area. A firm, flexible rectangle of a clear polythene (3 × 3 cm^2^) sheet was used to cover the wound after it had been marked with a fine-tipped permanent marker; the rats were then put back in their cages. Planimetrically, by converting the size of the wound on the transparent sheet into the weight of card paper with the same area, the area (mm^2^) within the boundaries of each trace was determined. Because the weight of the card paper per unit area was already known, estimating the weight of each card paper for a certain wound was simple. The wound area was measured on day 0, day 7, and 14 days post-wounding. Wilson’s formula was used to calculate the percentage of wound contraction [74].

$$\%\;\mathrm{Wound}\;\mathrm{contraction}=\frac{\mathrm{Day}\;0\;\mathrm{wound}\;\mathrm{area}-\mathrm{wound}\;\mathrm{area}\;\mathrm{on}\;a\;\mathrm{particular}\;\mathrm{day}\;\times 100}{\mathrm{Day}\;0\;\mathrm{wound}\;\mathrm{area}}$$

#### 3.6.4. Histopathology

Control and treated animals were sacrificed at the end of experimental period and tissues were removed from each animal’s wound site. Following sample fixation with 10% formalin, dehydration with ascending alcohol grades was performed. After being cleaned in xylene, the dehydrated samples were embedded in paraffin blocks and sectioned at 4–6 m thick. To examine the acquired tissue sections histopathologically using an electric light microscope, they were deparaffinized with xylol and stained with hematoxylin and eosin (H&E) [75]. 

### 3.7. Biochemical Analysis

#### 3.7.1. Measurement of Lipid Peroxidation

The level of malondialdehyde (MDA), as a marker of lipid peroxidation, was determined in the granulation tissue according to the kit’s instructions (Biodiagnostic, Egypt). The process depends on the interaction between thiobarbituric acid and MDA in an acidic solution at 95 °C for 30 min to produce a thiobarbituric acid reactive product; the pink product’s absorbance was then calculated at 534 nm [76].

#### 3.7.2. Estimation of Reduced Glutathione

The level of reduced glutathione was determined based on the kit’s instructions (Biodiagnostic, Egypt). The procedure is based on reducing GSH with 5,5′-dithiobis (2-nitrobenzoic acid) to produce a yellow reduced chromogen whose absorbance is directly proportional to the concentration of GSH and is calculated at 405 nm [77].

#### 3.7.3. Estimation of Reduced SOD

The level of superoxide dismutase (SOD) in the tissue was estimated according to the kit’s instructions (Biodiagnostic, Egypt). The methodology relies on the SOD’s capacity to prevent the reduction of the nitro-blue tetrazolium dye caused by phenazine methosulphate [78].

### 3.8. Statistical Analysis

All data were expressed as mean ± SEM and analysed by one-way ANOVA followed by Tukey’s post hoc test. All statistical analyses were performed using GraphPad Prism software (version 6.01). Probability values ≤ 0.05 were considered statistically significant.

### 3.9. Molecular Docking

The glycogen synthase kinase 3-β (GSK3-β), matrix metalloproteinases-8 (MMP-8) and nitric oxide synthase (iNOS) X-ray 3D structures were retrieved from the protein data bank (www.pdb.org), accessed on 12 October 2022 using the following IDs: 3F88, 5H8X and 3N2R, respectively [79,80,81]. Docking investigations were conducted utilising MOE 2019 [82], which was also utilised to develop the 2D interaction diagrams of docked ligands and potential targets. The fifteen identified major compounds (eight from liquorice and seven from *Sophora japonica*) were created with the default settings and saved in one MDB file. Each target’s active site was identified by the binding of the appropriate co-crystalized ligand. The co-crystalized ligand in each file was redocked in its corresponding binding site to validate the docking through calculating the RMSD values with the resulting docking poses (Supplementary Figures S1 and S2). The three enzymes’ active sites were docked with the MDB file, including all the main compounds, to complete the docking process. Triangular matcher and London *dg* were utilised as a placement method and scoring algorithm, respectively. The pharmacokinetic profiles of both glycyrrhizic acid and sophoraflavonoloside were computed using SWISS ADME (http://www.swissadme.ch/) (accessed on 10 March 2023).

### 3.10. Evaluation of Drug Interaction by CDI

The effect of drug combinations on the percentage of wound contraction, MDA, GSH and SOD levels was evaluated using the coefficient of drug interaction (CDI). For the reduced efficiency, the equation was CDI = AB/(A × B); and for the improved efficiency, the equation was CDI = (A × B)/AB, where AB is the ratio between the combination group and its control group; and A or B is the ratio between the single flavonoid fraction and its control group. The combination index scale was defined as follows in the current study: CDI *<* 0.9: synergistic, CDI = 0.9–1.1: additive and CDI *>* 1.1 antagonistic [4].

## 4. Conclusions

According to the findings of this study, the inclusion of *G. glabra* and *S. japonica* flavonoid-rich fractions in topical ointment preparation could efficiently accelerate wound closure rate. Additionally, they exerted strong antioxidant properties. Furthermore, the molecular docking studies of the identified major compounds provided a plausible mechanism prediction by which *G. glabra* and *S. japonica* flavonoid-rich fractions exert their wound healing effects. Liquiritin apioside and glycyrrhizic acid from *G. glabra* possessed higher affinities to the three target enzymes, GSK-3*ꞵ*, MMP-8 and iNOS. Similarly, sophoraflavonoloside and sophoraflavanone G, genistein 7,4′-di-O-*ꞵ*-D-glucopyranoside and kaempferol showed good energy binding scores with the target enzymes. Finally, this study suggested that using a combination of *G. glabra* and *S. japonica* could improve the healing of wounds. Future in-depth mechanistic research is still needed to verify these anticipated mechanisms of action.

## Figures and Tables

**Table 1:** Total phenolics and total flavonoids contents of *G. glabra* and *S. japonica* flavonoid-rich fractions.

Plant Name	TPC μg GA E/mg	TFC μg Quercetin E/mg
*G. glabra*	71.608 ± 3.23	46.99 ± 2.57
*S. japonica*	70.288 ± 1.94	49.91 ± 2.36

**Table 2:** Metabolite profiling of *Glycyrrhiza glabra* flavonoid-rich fraction via UPLC-ESI-MS in the negative and positive ion mode.

No.	*t_R_* (min)	Compound Name	[M − H] ^−^ (*m*/*z*)	[M + H] ^+^ (*m*/*z*)	Class	Molecular Formula	Ref.
1	8.58	Isoviolanthin	577.15	579.15	Flavonoid glycoside	C_27_H_30_O_14_	[21]
2	8.97	Liquiritin apioside	549.20	-	Flavonoid glycoside	C_26_H_30_O_13_	[21]
3	9.15	Liquiritin	417.25	-	Flavonoid glycoside	C_21_H_22_O_9_	[21]
4	10.15	Neolicuroside	549.20	-	Chalcone glycoside	C_26_H_30_O_13_	[21]
5	10.44	Licorice glycoside D2/D1	695.25	-	Flavonoid glycoside	C_35_H_36_O_15_	[21]
6	10.55	Isoliquiritin	417.15	419.15	Chalcone glycoside	C_21_H_22_O_9_	[40]
7	10.92	Liquiritigenin	255.10	-	Flavonoid aglycone	C_15_H_12_O_4_	[40]
8	11.07	Licorice glycoside E	692.20	-	Flavonoid glycoside	C_35_H_35_NO_14_	[21]
9	11.26	Licochalcone B	-	287.15	Chalcone	C_16_H_14_O_5_	
10	11.79	5,7-Dihydroxyflavanone	255.10	257.10	Flavonoid aglycone	C_15_H_12_O_4_	[21]
11	12.14	Licorice saponin G2	837.40	-	Triterpene	C_42_H_62_O_17_	[21]
12	12.48	Licorice saponin A3	983.45	-	Triterpene	C_48_H_72_O_21_	[21]
13	12.90	Echinatin	269.10	271.10	Chalcone	C_16_H_14_O_4_	[39]
14	13.66	Licorice saponin K2/H2	821.40	823.40	Triterpene	C_42_H_62_O_16_	[21]
15	14.12	Isoliquiritigenin	255.10	-	Chalcone	C_15_H_12_O_4_	[21]
16	14.40	Glycyrrhizic acid	821.35	-	Triterpene	C_42_H_62_O_16_	[21]
17	14.87	Glycyrrhisoflavanone	369.20	371.20	Isoflavanone	C_21_H_20_O_6_	[41]
18	15.51	Glabrene	-	323.20	Isoflavene	C_20_H_18_O_4_	[21]
19	16.08	Licochalcone D	353.20	355.20	Chalcone	C_21_H_22_O_5_	[21]
20	16.26	Glabranin	323.20	-	Flavonoid aglycone	C_20_H_20_O_4_	[24]
21	16.92	Licorisoflavan A	-	439.10	Isoflavan	C_27_H_34_O_5_	[21]
22	17.07	Glycocoumarin	367.10	-	Coumarin	C_21_H_20_O_6_	[21]
23	17.79	Kanzonol H	423.15	-	Isoflavan	C_26_H_32_O_5_	[21]
24	18.12	3-Hydroxyglabrol	407.20	409.20	Flavonoid aglycone	C_25_H_28_O_5_	[21]
25	18.54	Glabridin	323.20	325.20	Isoflavane	C_20_H_20_O_4_	[21]
26	19.23	Kanzonol Y	409.20	411.25	Dihydrochalcone	C_25_H_30_O_5_	[21]
27	19.47	Glabrol	391.25	393.25	Flavonoid aglycone	C_25_H_28_O_4_	[21]
28	20.28	Licoflavanone	339.10	-	Flavonoid aglycone	C_20_H_20_O_5_	[24]
29	20.47	Isolicoflavonol	-	355.20	Flavonoid aglycone	C_25_H_27_O_4_	[41]
30	21.93	Hydroxy-oleic acid	297.30	-	Fatty acid	C_18_H_34_O_3_	[42]
31	22.08	Licochalcone A	-	339.20	Chalcone	C_21_H_22_O_4_	[41]
32	23.56	Glycyrrhetinic acid	469.20	471.35	Triterpene	C_30_H_46_O_4_	[21]

**Table 3:** Metabolite profiling of *Sophora japonica* flavonoid rich fraction via UPLC-ESI-MS in the negative and positive ion mode.

No.	*t_R_* (min)	Compound Name	[M − H] ^−^ (*m*/*z*)	[M + H] ^+^ (*m*/*z*)	Class	Molecular Formula	Ref.
1	3.57	1,3,5-Tri-*O*-caffeoylquinic acid	677.25	-	Phenylpropanoids	C_34_H_30_O_15_	[48]
2	5.04	Quercitrin (Quercetin-3-O-L-rhamnoside)	447.10	-	Flavonoid glycoside	C_21_H_20_O_11_	[31]
3	7.50	Kaempferol 3-O-*α*-l-rhamnopyranosyl(1→6) -*β*-d-glucopyranosyl(1→2)-*β*- D -glucopyranoside-7-O- *α* -l-rhamnopyranoside	901.25	-	Flavonoid tetra glycoside	C_39_H_50_O_24_	[33]
4	7.91	Sophoraflavanone G	-	425.20	Flavonoid aglycone	C_25_H_28_O_6_	[47]
5	8.23	Genistein 7-*O*-*β*-D-glucopyranoside-4′-*O*-[(*ꞵ*-D-glucopyranosyl)- (1→2)- *ꞵ*-D-glucopyranoside]	755.25	-	Isoflavonoid glycoside	C_33_H_40_O_20_	[44]
6	8.49	Genistein 7-O-*ꞵ*-D-glucopyranoside-4′-O- [(*α* -L-rhamnopyranosyl)-(1→2)- *ꞵ*-D-glucopyranoside]	739.20	-	Flavonoid glycoside	C_33_H_39_O_19_	[44]
7	8.81	Sophoraflavonoloside	609.20	611.20	Flavonoid glycoside	C_27_H_30_O_16_	[43]
8	9.13	Genistein 7,4′-di-O-*ꞵ*-D-glucopyransoide	593.10	-	Isoflavonoid glycoside	C_27_H_30_O_15_	[43]
9	9.53	Paratensein-7-*O*-glucoside	461.15	-	Flavonoid glycoside	C_22_H_22_O_11_	[38]
10	9.69	Narcissin (Isorhamnetin-3-O-rutinoside)	463.20	-	Flavonoid glycoside	C_28_H_32_O_16_	[28]
11	10.00	Myricetin-O-coumaroyl- glucoside	625.35	-	Flavonoid glycoside	C_30_H_26_O_15_	[42]
12	10.19	Kaempferitrin	577.20	579.20	Flavonoid glycoside	C_27_H_30_O_14_	[28]
13	10.73	Quercetin 3-O-*ꞵ*-D-glucopyranoside	463.25	-	Flavonoid glycoside	C_21_H_19_O_12_	[37]
14	10.96	Sophorabioside	577.20	-	Isoflavonoid glycoside	C_27_H_30_O_14_	[43]
15	11.16	Nepetin 4′-glucoside	477.50	-	Flavonoid glycoside	C_22_H_22_O_12_	[48]
16	12.25	Quercetin	301.20	-	Flavonoid	C_15_H_10_O_7_	[46]
17	12.41	Alopecurone A	649.45	-	Flavonostilbene	C_39_H_38_O_9_	[49]
18	12.96	Kurarinone	-	439.50	Flavonoid aglycone	C_26_H_30_O_6_	[47]
19	13.21	Genistein	269.45	-	Isoflavonoid	C_15_H_10_O_5_	[45]
20	14.57	Apigenin	267.50	269.50	Flavonoid aglycone	C_15_H_10_O_5_	[31]
21	16.67	Kaempferol	283.10	285.10	Flavonoid aglycone	C_15_H_10_O_6_	[45]
22	19.70	Tamarixetin	-	317.25	Flavonoid aglycone	C_16_H_12_O_7_	[46]
23	21.16	Medicagol	295.25	-	Coumestans	C_16_H_8_O_6_	[50]

**Table 4:** Wound healing processes score of different treatment groups on day 14.

Group NO.	Group	Thickness of Epithelial Cells	Inflammatory Cells	Collagen
I	Negative control (Wound)	+ + + + +	+ + + + +	+
II	Plain gel	+ + + + +	+ + + + +	+ +
III	Plain ointment	+ + + +	+ + + + +	+ + +
IV	Positive control (Nolaver^®^)	+ +	+ +	+ + + +
V	Ointment of *G. glabra* and *S. japonica* combination (1:1) (ABO)	+	+	+ + + +
VI	Gel of *G. glabra* and *S. japonica* combination (1:1) (ABG)	+ +	+ + +	+ + +
VII	Ointment of *G. glabra* (AO)	+ +	+ + +	+ + +
VIII	Ointment of *S. japonica* (BO)	+ +	+	+ + +
IX	Gel of *G. glabra* (AG)	+ + +	+ +	+ +
X	Gel of *S. japonica* (BG)	+ + + +	+ + +	+ +

HE (Hematoxylin and eosin) stained sections were scored as mild (+), moderate (+ + +) and severe (+ + + + +) for epidermal and/or dermal remodelling.

**Table 5:** Nature of interaction between *G. glabra* and *S. japonica* flavonoid-rich fractions as determined by CDI.

Parameter	CDI	Effect of Ointment Combination	CDI	Effect of Gel Combination
Percent of wound contraction on day 7	0.32	Synergistic	0.30	Synergistic
Percent of wound contraction on day 14	0.32	Synergistic	0.27	Synergistic
MDA level	0.37	Synergistic	0.70	Synergistic
GSH level	0.86	Synergistic	0.71	Synergistic
SOD level	0.61	Synergistic	0.78	Synergistic

**Table 6:** The docking scores obtained by the major compounds identified in *G. glabra* and *S. japonica* against the three target enzymes GSK-3*ꞵ*, MMP-8 and iNOS.

Major Identified Compounds in *G. glabra*			
Compound	Docking Scores Kcal/mol		
	GSK-3*ꞵ* 3F88	MMP-8 5H8X	iNOS 3N2R
Co-crystalized ligand	3HT (−15.7)	7FY (−13.2)	XJH (−16.4)
Isoliquiritigenin	−12.0	−10.2	−11.4
Liquiritin apioside	−14.1	−12.8	−14.5
Neolicuroside	−13.6	−15.4	−13.5
Kanzonol Y	−13.4	−11.3	−11.2
Glabridin	−12.8	−10.8	−10.7
Glabrol	−11.9	−10.3	−13.2
Glycyrrhizic acid	−15.2	−11.9	−18.2
Glycyrrhetinic acid	−11.3	−10.5	−11.1
Major identified compounds in *Sophora japonica*			
Compound	Docking scores Kcal/mol		
	GSK−3*ꞵ* 3F88	MMP−8 5H8X	iNOS 3N2R
Kaempferol	−13.1	−13.7	−11.6
Sophoraflavonoloside	−14.3	−13.4	−16.1
Sophoraflavanone G	−13.5	−10.4	−14.6
Genistein 7,4′-di-O-*ꞵ*-D-glucopyransoide	−16.9	−9.9	−13.4
Genistein	−10.5	−11.4	−11.2
Tamarixetin	−13.1	−9.9	−10.6
Kurarinone	−11.6	−12.3	−12.3

**Table 7:** Composition of the prepared ointment formulation.

Ingredients	Weight (g)
Wool fat	50
Hard paraffin	50
Cetostearyl alcohol	50
White soft paraffin	850
	1000

## Data Availability

Data are contained within the article.

## Supplementary Materials

The following supporting information can be downloaded at: https://www.mdpi.com/article/10.3390/molecules28072994/s1, Figure S1. Docking validation of the three targets (A) GSK3- *β* (PDB ID: 3F88), (B) MMP-8 (PDB ID: 5H8X) and (C) iNOS (PDB ID: 3N2R), Figure S2. Interaction of the three co-crystallized ligands (A) GSK3- *β* (PDB ID: 3F88), (B) MMP-8 (PDB ID: 5H8X) and (C) iNOS (PDB ID: 3N2R).
