# Supplementary material for: Synergistic Effect of Sophora japonica and Glycyrrhiza glabra Flavonoid-Rich Fractions on Wound Healing: In Vivo and Molecular Docking Studies
Source: Molecules. 2023 Mar 27;28(7):2994. doi: 10.3390/molecules28072994 (PMC10096052; doi:10.3390/molecules28072994)
Supplement: Supplementary file 1 [file molecules-28-02994-s001.zip › molecules-2269229-supplementary.pdf]

Synergistic effect of *Sophora japonica* and *Glycyrrhiza glabra* Flavonoid-Rich Fractions on Wound Healing: *In vivo* and Molecular Docking Studies

Shaza H. Aly [1, *], Ahmed M. Elissawy [2,3], Abdallah Mahmoud [4], Fatma Sa'eed El-Tokhy [5], Sherif S. Abdel Mageed [4], Mahmoud A. El Hassab [6], Wagdy M. Eldehna [7,8], Hadia Almahli [9], Sara T. Al-Rashood [10], Faizah A. Binjubair [10] and Abd El-Nasser B. Singab [2,3,*]

[1] Department of Pharmacognosy, Faculty of Pharmacy, Badr University in Cairo (BUC), Badr City, Cairo 11829, Egypt

[2] Department of Pharmacognosy, Faculty of Pharmacy, Ain-Shams University, 11566, Cairo, Egypt

[3] Center of Drug Discovery Research and Development, Ain Shams University, Cairo, Egypt

[4] Pharmacology and Toxicology Department, Faculty of Pharmacy, Badr University in Cairo (BUC), Badr city, Cairo, 11829, Egypt

[5] Department of Pharmaceutics and pharmaceutical technology, Badr University in Cairo (BUC), Badr city, Cairo, 11829, Egypt

[6] Department of Medicinal Chemistry, Faculty of Pharmacy, King Salman International University (KSIU),
South Sinai 46612, Egypt

[7] Department of Pharmaceutical Chemistry, Faculty of Pharmacy, Kafrelsheikh University, Kafrelsheikh 33516, Egypt

[8] School of Biotechnology, Badr University in Cairo, Badr City, Cairo 11829, Egypt

[9] Department of Chemistry, University of Cambridge, Cambridge CB2 1EW, UK

[10] Department of Pharmaceutical Chemistry, College of Pharmacy, King Saud University, P.O. Box 2457, Riyadh 11451, Saudi Arabia

* Correspondence: (A.N.B.S.) dean@pharma.asu.edu.eg, (S.H.A.) shaza.husseiny@buc.edu.eg

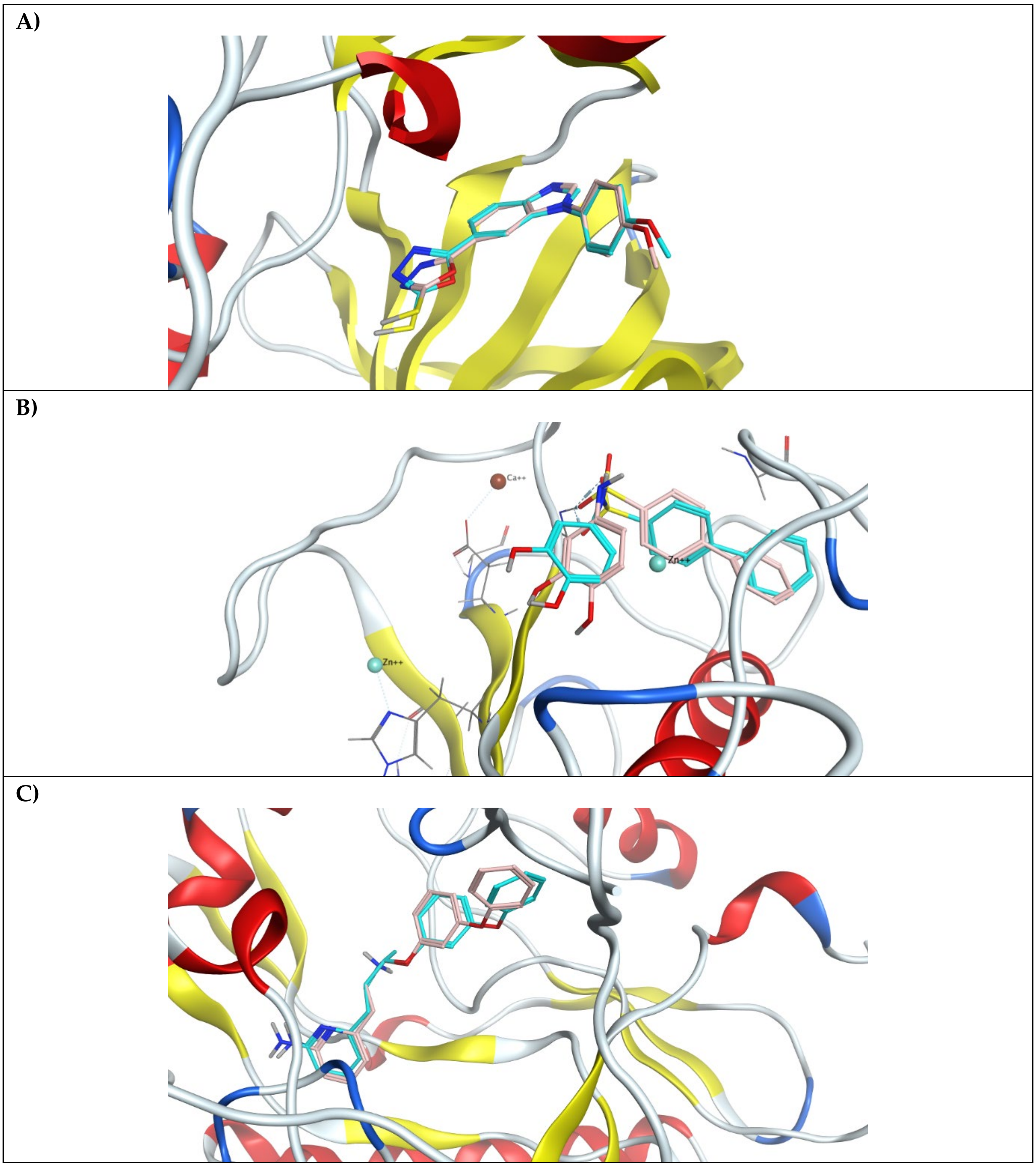

Figure S1. Docking validation of the three targets **A)** GSK3- β (PDB ID: 3F88), **B)** MMP-8 (PDB ID: 5H8X) and **C)** iNOS (PDB ID: 3N2R).

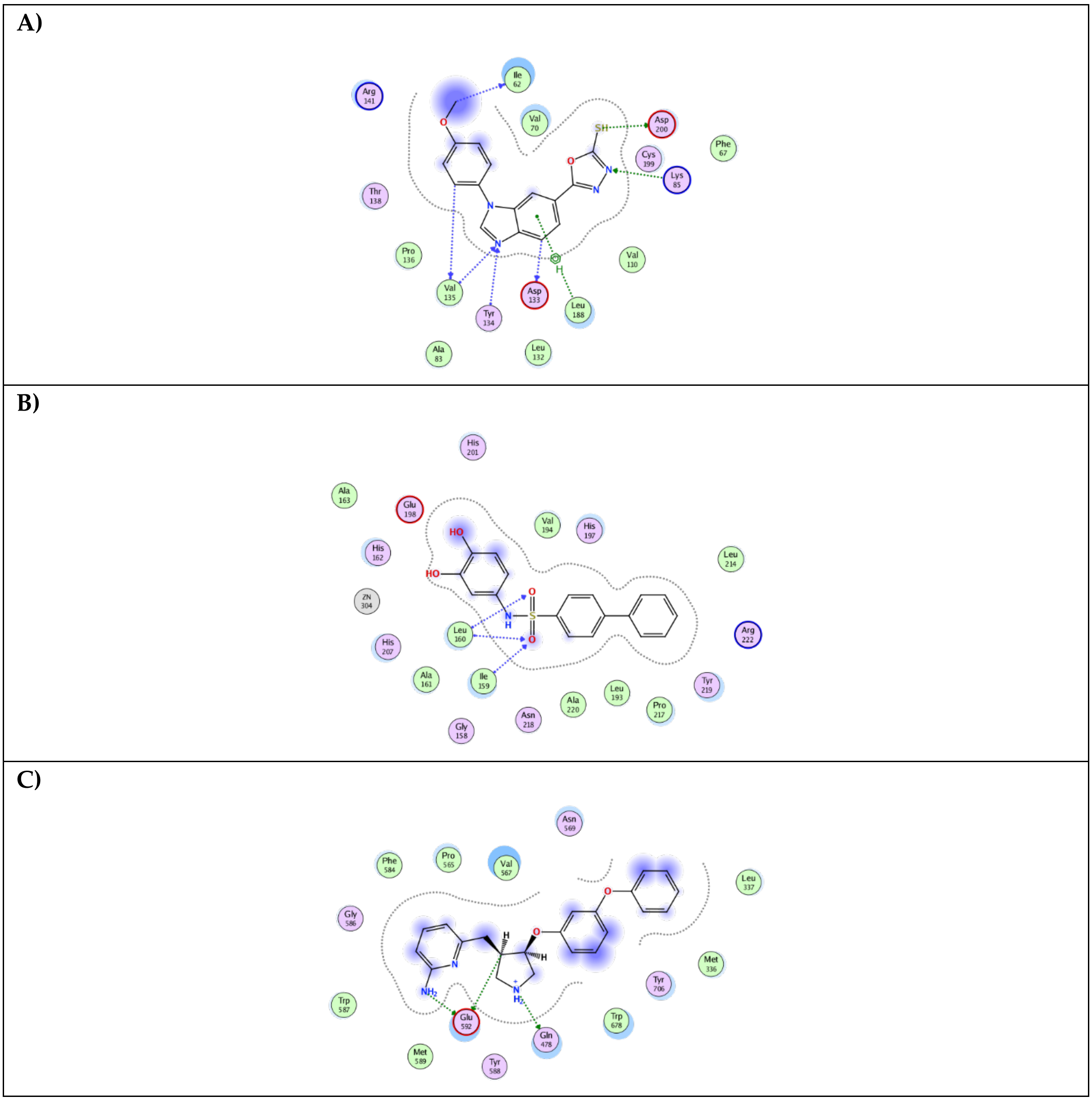

Figure S2. Interaction of the three co-crystallized ligands with **A)** GSK3- β (PDB ID: 3F88), **B)** MMP-8 (PDB ID: 5H8X) and **C)** iNOS (PDB ID: 3N2R).
